# Evolution of the holozoan ribosome biogenesis regulon

**DOI:** 10.1186/1471-2164-9-442

**Published:** 2008-09-24

**Authors:** Seth J Brown, Michael D Cole, Albert J Erives

**Affiliations:** 1 Department of Genetics, Dartmouth Medical School, 1 Medical Center Drive, Lebanon, NH 03756, USA; 2 Department of Pharmacology and Toxicology, Dartmouth Medical School, 1 Medical Center Drive, Lebanon, NH 03756, USA; 3 Department of Biological Sciences, Dartmouth College, Hanover, NH 03755, USA

## Abstract

**Background:**

The ribosome biogenesis (RiBi) genes encode a highly-conserved eukaryotic set of nucleolar proteins involved in rRNA transcription, assembly, processing, and export from the nucleus. While the mode of regulation of this suite of genes has been studied in the yeast, *Saccharomyces cerevisiae*, how this gene set is coordinately regulated in the larger and more complex metazoan genomes is not understood.

**Results:**

Here we present genome-wide analyses indicating that a distinct mode of RiBi regulation co-evolved with the E(CG)-binding, Myc:Max bHLH heterodimer complex in a stem-holozoan, the ancestor of both Metazoa and Choanoflagellata, the protozoan group most closely related to animals. These results show that this mode of regulation, characterized by an E(CG)-bearing core-promoter, is specific to almost all of the known genes involved in ribosome biogenesis in these genomes. Interestingly, this holozoan RiBi promoter signature is absent in nematode genomes, which have not only secondarily lost Myc but are marked by invariant cell lineages typically producing small body plans of 1000 somatic cells. Furthermore, a detailed analysis of 10 fungal genomes shows that this holozoan signature in RiBi genes is not found in hemiascomycete fungi, which evolved their own unique regulatory signature for the RiBi regulon.

**Conclusion:**

These results indicate that a Myc regulon, which is activated in proliferating cells during normal development as well as during tumor progression, has primordial roots in the evolution of an inducible growth regime in a protozoan ancestor of animals. Furthermore, by comparing divergent bHLH repertoires, we conclude that regulation by Myc but not by other bHLH genes is responsible for the evolutionary maintenance of E(CG) sites across the RiBi suite of genes.

## Background

Ribosome biogenesis (RiBi) is a primary function of the nucleolus [1-3]. In the nucleolus, rRNA molecules are synthesized as precursors by DNA-directed RNA Pol I and Pol III. Nascent rRNAs then undergo extensive chemical modifications and RNA cleavage reactions. Numerous RiBi proteins are involved both in this enzymatic processing as well in assisting with proper rRNA folding to produce functional ribosomal subunits [1]. Synthesizing functional ribosomes requires immense coordination because gene products from all three DNA-dependent RNA polymerases are required to ensure proper stoichiometry of ribosomal components. This level of co-regulation is likely to be controlled through a highly-specific DNA signature as seen in other gene regulatory systems [4]. Such a signature would allow the appropriate factors to co-ordinately regulate the RiBi genes as a distinct regulon. For example, in the yeast *Saccharomyces cerevisiae *two important regulatory motifs consisting of the PAC (polymerase A and C) and RRPE (Ribosomal RNA Processing Element) motifs have been identified in RiBi genes [5-7]. In animals no known factor or motif is known to coordinate the entire RiBi set. However, the metazoan transcription factor Myc has been found to target at least some RiBi genes.

Myc is a bHLH DNA-binding protein present in animals where it plays an important role in cell growth and proliferation by regulating gene expression during development and tumorigenesis [8-10]. Myc functions by heterodimerizing with its obligate partner Max to bind the sequence 5'-CACGTG [a CG-core E-box, or E(CG)] and transactivate target genes [11-14]. Previous studies have implicated the Myc transcription factor in rRNA transcription [15-17], but the possibility that Myc could directly regulate the hundreds of gene products involved in RiBi, as opposed to a few early genes or key steps [18-20], has not been investigated. Also unknown is when such an RiBi circuit or other non-RiBi targets of Myc may have evolved and whether it was in the most recent common ancestor of Bilateria (protostomes and deuterostomes), Metazoa (animals), Holozoa (Metazoa + choanoflagellates), Opisthokonta (Holozoa + fungi), or Eukaryota.

Here, we use a multi-genomic approach to show that the vast majority of genes implicated in ribosome biogenesis are associated with E(CG)-bearing core promoters in all holozoan genomes containing Myc, and thus constitutes a uniquely holozoan RiBi regulon. Max, Mad, and Mnt, all members of the Myc bHLH superfamily, are all either insufficient or dispensable in explaining the correlation of E(CG) with RiBi as revealed by a comparison of multiple eukaryotic genomes, which differ in their bHLH repertoires. Thus, in addition to known RiBi targets of Myc [18-20] and the similar growth defects of both RiBi and myc mutant alleles [8-10,18,20,21], our comparative genomic results suggest that the characteristic RiBi E(CG) core promoter architecture co-evolved with a proto-Myc:Max complex in a unicellular holozoan ancestor. This is consistent with the metabolic evolution of a unique, Myc-Max regulated, RiBi growth signalling pathway in an ancient unicellular heterotroph.

## Results and discussion

### Overall approach

To gain insight into the regulatory control of Ribosome Biogenesis (RiBi) in animals, we first wanted to characterize a co-regulated RiBi gene set definable by a shared regulatory signature, a component of which would be the Myc-Max binding site E(CG). *A priori*, we had no reason to expect whether a common regulatory signature would be found across the large number of known ribosome biogenesis genes or whether such a signature would be limited to only a subset (i.e. the *sensitivity *of the postulated signature for RiBi genes). Furthermore, we set no expectation on whether this signature would necessarily be present or absent in other non-RiBi genes devoted to cell growth or other functions (i.e. the *specificity *of the postulated signature for RiBi genes). For this purpose, we chose the *Drosophila melanogaster *genome because of its relatively compact size and absence of genome wide duplications characteristic of vertebrates. Both of these properties facilitate the identification of a sequence signature and the characterization of its sensitivity and specificity for entire biological functions because they simplify the ability to conduct and interpret computational queries of genome sequence.

Having identified the list of genes in this fly regulon we would then utilize the many available eukaryotic genome sequences to identify shared sequence signatures associated with this regulon in each genome. Because of the highly conserved nature of the protein functions associated with ribosome biogenesis, it is likely that this list of genes would remain co-regulated in other genomes despite evolution of the regulatory signatures associated with this regulon.

### E(CG) is highly specific to the fly RiBi regulon

An overwhelming majority of confirmed Myc targets contain E(CG) near the transcriptional start site (TSS) [19]. We therefore examined all fly genes with promoter proximal E(CG) sites to determine whether they were related by a common cellular function. We searched the *Drosophila melanogaster *genome and identified only 390 promoter sequences containing an E(CG) site in the core promoter region (160 bp centered around +1) out of 20,468 annotated transcripts (14,752 genes). Remarkably, these genes include most proteins known to be involved throughout ribosome biogenesis [see Additional file 1]. To examine the relationship between the E(CG) motif and ribosome biogenesis in more detail, we used Gene Ontology (GO) classification of the yeast genome to map 121 fly orthologs of yeast nucleolar genes. Of these, ~75% possess E(CG), indicating that the majority are under control of a common regulatory motif in *Drosophila *[see Additional file 1]. As explained below, many additional fly E(CG)-bearing genes, which are not conserved as orthologs in yeast, are likely to be related to ribosome biogenesis. Furthermore, as detailed by multiple statistical tests conducted in this study, the rate of E(CG) motifs in the *Drosophila *RiBi gene promoters is significantly elevated relative to promoters of genes not known to be involved in nucleolar functions and/or ribosome biogenesis.

### E(CG)-bearing, RiBi-type promoters are unique

Any examination restricted to gene orthologs precludes the identification of genes not conforming to a 1-to-1 orthology. To this end, it would be ideal to search the entire genome to provide a comprehensive analysis of the entire E(CG) fly RiBi regulon. This method could identify novel fly RiBi genes harboring E(CG) sites independent of a 1-to-1 orthology and ambiguous annotations of transcriptional initiation sites. To carry out this whole genome query, we first searched for additional motifs that comprised the full core promoter context of RiBi genes. With additional motifs in hand, highly specific whole genome queries for promoter-linked E(CG) sites could be achieved, thereby identifying a more complete RiBi regulon.

Myc's known co-localization to core promoters [16] suggests it may define or associate with a distinct core promoter architecture. We therefore looked for novel elements that may be specific to RiBi promoters but infrequent across other promoters. We also investigated whether promoters bearing E(CG) are associated with specific core promoter elements such as TATA-boxes, Initiator sequences, downstream promoter elements (DPEs), and other common motifs [22]. As detailed below, these results support the idea that the fly RiBi-type promoter, uniquely characterized by the E(CG) site, defines a distinctive and highly specific promoter architecture, which is useful in identifying this gene set in *Drosophila*.

**Figure 1:**
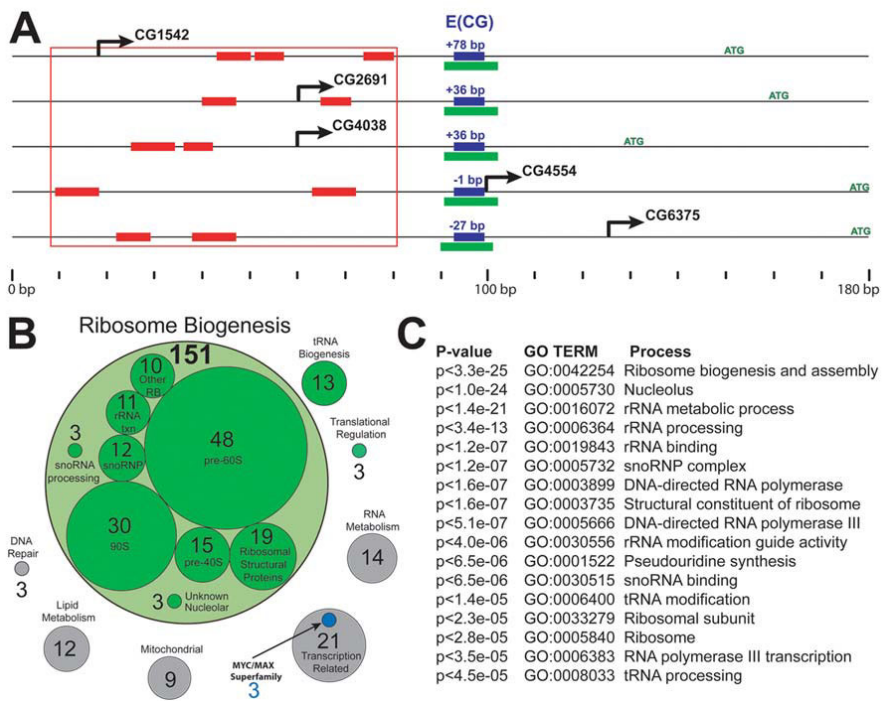
**E(CG) is a core promoter element of Drosophila ribosome biogenesis (RiBi) genes**. (A) Fly RiBi genes (5 examples shown) generally possess three common features around the transcriptional start site (rightward pointing arrow) and upstream of the translational start (ATG). This distinct promoter architecture is characterized by a CG-core E-box (blue box), a specific E(CG) flanking motif (green box) and a coordinating cluster of sites matching the DNA Replication Element, DRE, (red boxes) spanning a distance less than 100 bp. The distance of E(CG) to the TSS for each gene is indicated above E(CG). G6375 corresponds to the *pit *gene, which is a known Myc target and an RiBi gene [18]. (B) This core promoter architecture identifies several functional groups of genes associated with RiBi (green circles). The number of genes is indicated for functional groups with more than 3 members. The sum of 151 genes (large circle) is the sum of all of the individual subfunctions with specific roles in Ribosome Biogenesis. The RiBi genes encode a variety of domains and protein folds including RNA-binding regions (RNP-1), C-terminal helicases, DEAD/DEAH box helicases, WD-40 repeats, ARM repeats, Histone-folds, AAA ATPases and many others [see Additional file 1]. (C) The results of genome queries in *Drosophila *for E(CG) type core promoters results in a highly significant enrichment of GO terms directly related to ribosome biogenesis (nucleolar, rRNA metabolism, rRNA binding, snoRNA complex, pseudouridine synthesis, ribosomal subunits, etc.)

By searching for novel motifs, we first noted that the flanks of the E(CG) site often match an extended consensus MAACACGTGYG (M = A/C, Y = C/T). Three out of every four core promoters that contain this extended E(CG) consensus map to RiBi genes (Fig. 1A). As a negative control for the specificity of the flanking sequence, we also searched the entire genome with an E(CG) motif in which the flanking pattern was maximally divergent from the observed, RiBi-specific, CG-core E-box flanking pattern. This "anti-flank" E(CG) motif, KKYCACGTGRMK (K = G/T, R = A/G), maps to almost 3-fold more sites than the extended E(CG) consensus, but nonetheless is absent from the core promoters of known nucleolar or RiBi orthologs (data not shown).

We also identified sequences corresponding to the DRE core promoter element, 5'-CTATCGATA, as previously reported [23]. DRE (DNA-replication element) binds DREF (DRE factor) and is associated with promoters involved in DNA replication [24]. We observe a cluster of DRE sites in many RiBi gene promoters, where it occurs immediately upstream of E(CG) (Fig. 1A). When the fly genome is queried for regions containing at least 2 DRE motifs and an E(CG) within an 80 bp window, this highly specific signature identifies 126 loci in the genome. Over half of these are known to be associated with RiBi [see Additional file 1]. Tellingly, DRE motifs are not associated with E(CG) sites containing the anti-flank sequence (i.e. KKYCACGTGRMK).

This analysis provides enough information to distinguish most core-promoter linked E(CG) sites from the majority of ~15,000 E(CG) sites that lie outside of promoter regions and likely represent random background occurrences. We queried the entire *Drosophila *genome for the different E(CG)-type core promoter signatures [E(CG) + flanks, or E(CG) + DRE, or E(CG) + mapped 5'-end]. The largest functional group of genes identified across the genome is the RiBi gene set (151/321 genes; Fig. 1). These genes are involved at all steps of ribosomal processing including factors involved in rRNA transcription (11 genes), snoRNA processing (3 genes), snoRNPs (12 genes), 90S particles (30 genes), pre-60S particles (48 genes), 40S particles (15 genes), ribosome structure (19 genes), unknown steps in ribosome biogenesis (10 genes), and unknown functions of the nucleolus (3 genes). Some of these RiBi genes have previously been implicated as potential direct or indirect Myc targets [16,18,19,23]. Some genes encode products that are only known to be localized to the nucleolus (e.g. spermidine synthase) [16]. Others participate in tRNA modification in addition to rRNA modification [25,26]. Altogether these results support the hypothesis that the many RiBi genes are co-regulated and identifiable through a specific E(CG)-bearing promoter architecture.

### Characterization of the RiBi regulon across Eukaryota

**Figure 2:**
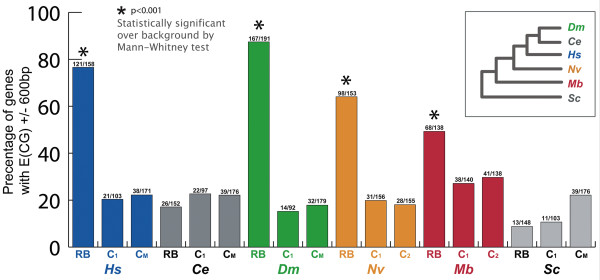
**Holozoan RiBi promoters are enriched in E(CG) sites**. The percentage of E(CG)-bearing promoters in RiBi genes is two to four fold higher in *D. melanogaster *(Dm), *H. sapiens *(Hs), *N. vectensis *(Nv), and *M. brevicollis *(Mb) relative to negative control sequences composed of promoter regions of downstream conserved genes (*C*_1_), the 3' regions of RiBi genes (C2), or the promoters of genes with GO mitochondrial classification (*C*_*M*_). This difference between RiBi and *C*_1_, *C*_2_, or *C*_*M *_is lacking in outgroup genomes such as *S. cerevisiae *(Sc), which lack Myc, as well as in the nematode genome of *C. elegans *(Ce), which has secondarily lost Myc (Fig. 3). Inset depicts phylogenetic relationships among these organisms.

To determine the evolutionary origins of the RiBi regulon, we first measured the conservation of E(CG) motifs in the RiBi genes of other bilaterian genomes, including humans and nematodes. Of the human RiBi genes, 77%, possess an E(CG) in a window ± 600 bp from the 5' annotated end, which is a significantly elevated compared to the ~20% background level in control promoters (Fig. 2; Table 1). In contrast with humans, there is no elevated level of E(CG) in RiBi genes in the nematode genome of *C. elegans *despite the conservation of such genes (Fig. 2). The presence of an E(CG)-RiBi signature in both a deuterostome (humans) and a protostome (flies) suggests that the absence in another protostome (nematodes) is a secondary loss.

**Table 1:** Highly conserved genes with an E(CG)-bearing promoter.

**A – Human genes with E(CG)-bearing promoters across bilaterian orthologs**			
ABCF1*	ESF1*	NOL11*	RIOK1*
ATHL1	GNL2*	NOL14*	RPL11*
BXDC1*	GRWD1*	NOL5_HUMAN*	RPL8*
BXDC5*	HEATR1*	NOL5A*	RRP12*
BYSL*	HTF9C_HUMAN*	NOL6*	RRP9*
C1orf107*	IMP4*	NOLA1*	RRS1*
C1orf181*	KAD6_HUMAN*	NP_060822.2*	SDAD1*
C6orf66	KIAA0020*	NP_079425.3	SRM*
CCDC86*	KIAA0409*	PAQR3	SURF6*
CEBPZ*	KLHDC4	PFAS	TIMM10
CIRH1A*	LYAR*	PNO1*	TRMT1*
DDX18*	MBIP	POLR1B*	UMPS
DDX51*	MEN1	POLR1E*	UTP20*
DDX52*	MRTO4*	POLRMT	WDR3*
DDX54*	MYBBP1A*	PRMT3	WDR36*
DHX37*	NAT10*	PWP1*	WDR75*
DPH5	NHP2L1*	PWP2*	WDR89
DUS3L*	NLE1*	QTRTD1*	
EBAG9	NM_005452*	RBM19*	
ELP3	NOL1*	RCL1*	
**B – Human genes with E(CG)-bearing promoters across holozoan orthologs**			
ATIC	EBNA1BP2*	MRTO4*	PRMT3*
BMS1L*	ESF1*	NAT10*	PWP1*
BXDC1*	EXOSC1*	NHP2L1*	PWP2*
BXDC5*	FARSLB*	NLE*	SLC25A32
BYSL*	GTPBP9	NOL14*	SRM*
DDX4	KAD6_HUMAN*	NOP5*	TRMT1*
DDX54*	KLF15	PNO1*	VPS29
DHX37*	LYAR*	POLR3K*	
DPH5	MCCC2	PRDX4	

(A) A list of all human genes with orthologous fly genes containing an E(CG) tightly linked to the core promoter in both orthologs. These genes have an E(CG) ± 600 bp and ± 80 bp from the transcriptional initiation site in the pair of human and fly orthologs, respectively. Genes with asterisks are known to be involved in ribosome biogenesis. See Additional file 1 for a more exhaustive list of genes described in this study. (B) A list of all human genes that have been evolutionarily maintained with E(CG)-bearing promoters across orthologous holozoan loci (humans, flies, cnidarians, and choanoflagellates). These genes have an E(CG) ± 600 bp from the annotated 5'-end in each genome. Missing from this list are genes that may be E(CG)-bearing in a genome but are not yet annotated by full-length transcripts. Genes with asterisks are known to be involved in ribosome biogenesis. See Additional file 1 for a more exhaustive list of genes described in this study.

**Figure 3:**
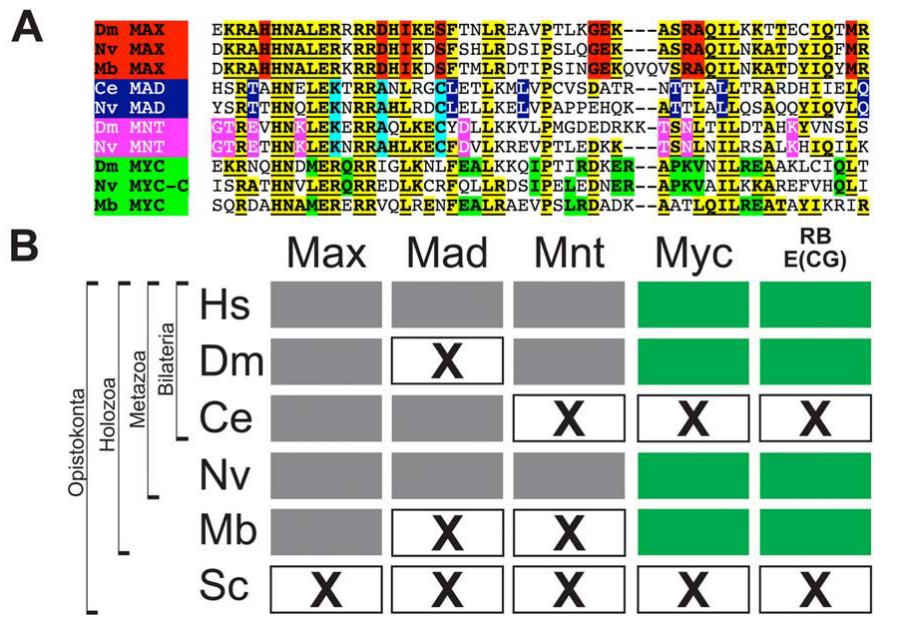
**The RiBi-E(CG) regulon occurs only in Myc-bearing holozoan genomes**. (A) Specific amino acid residues in holozoan MAX (red), MAD (blue), MNT (pink), and MYC (green) allow identification among the MYC/MAX superfamily bHLH genes (common superfamily residues in yellow and underlined). Only three bHLH genes were found in the choanoflagellate genome of *Monosiga brevicollis*: Mb-MYC, MbMAX and MbMUSH, corresponding to Myc and Max orthologs, and a distant MITF/USF/SREBP homolog (not shown). No Myc and Max orthologs were found outside of Holozoa. The predicted amino acid sequences of the bHLH regions of the *M. brevicollis *Myc/Max family of genes are shown aligned to *Drosophila*, *Caenorhabditis*, and *Nematostella *orthologs. (B) The presence of Myc (green filled boxes) is correlated with multiple genomes possessing the E(CG)-RiBi signature (green filled boxes). Other bHLH genes in the Myc superfamily (Max, Mad, Mnt; gray filled boxes) are either not necessary (Mad or Mnt) or insufficient (Max) to explain the occurrence of E(CG) sites in the RiBi regulon. "X" boxes indicate absence of a gene or E(CG) signature as indicated.

To test whether components of the fly RiBi regulon are conserved outside of Bilateria, we analyzed the presence of the E(CG) signature in the RiBi orthologous cohort in the genomes of more distantly-related organisms (Figs. 2 and 3). For a metazoan outgroup to Bilateria, we used the cnidarian genome of *Nematostella vectensis *[27]. For a holozoan outgroup to Metazoa, we used the choanoflagellate genome of *Monosiga brevicollis *[28]. For an opisthokont out-group to Holozoa, we used the baker's yeast genome of *Saccharomyces cerevisiae*.

We identified all of the RiBi orthologs between the fly, human, and yeast genomes and their apparent orthologs in the *Nematostella *and *Monosiga *genomes and were able to find at least 100 such genes in each genome. We found that each holozoan genome (except *C. elegans*) possesses E(CG) sites across ~50% to 90% of its identifiable RiBi genes in the region ± 600 bp from the 5'-most end (Fig. 2). This corresponds to a statistically significant (p < 0.001) two-fold to four-fold elevated level of E(CG) relative to the core promoter regions of adjacent control genes (*C*_1 _in Fig. 2). As additional negative controls, we analyzed the frequency of E(CG) in the region ± 600 bp from the 3' end of the same test genes (*C*_2_), or in the promoters of genes with mitochondrial GO terms (*C*_*M*_). We again found no enrichment over background (Fig. 2).

**Figure 4:**
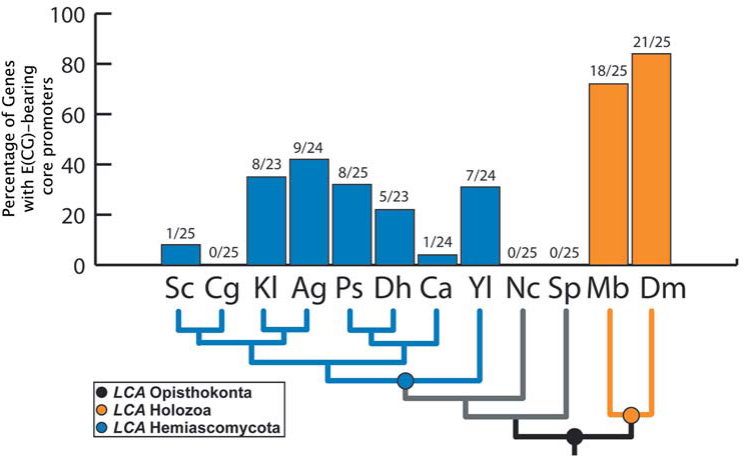
**Frequency of E(CG) in opisthokont RiBi promoters**. The frequency of E(CG) in 25 opisthokont RiBi orthologs was investigated. These 25 RiBi orthologs were selected based on the presence of conserved E(CG) sites in the human, fly, sea anemone, and choanoflagellate orthologs (Table 1B). DNA sequences 500 bp upstream from the translational start sites in the RiBi orthologs of *S. cerevisiae *(Sc), *C. glabrata *(Cg), *K. lactis *(Kl), *A. gossypii *(Ag), *P. stipitis *(Ps), *D. hansenii *(Dh), *C. albicans *(Ca), *Y. lipolytica *(Yl), *N. crassa *(Nc), *S. pombe *(Sp), and *M. brevicollis *(Mb) were collected. For *D. melanogaster *(Dm), 500 bp of DNA sequence (± 250 bp) from the 5' annotated end was collected. The sequences of each of these opisthokont promoters was analyzed for the presence of E(CG) motifs. The percentage of (ECG) in each species' RiBi orthologs is depicted on the Y-axis with the number of orthologs containing E(CG) over the total ortholog number of orthologs displayed above each genome. Key nodes for latest common ancestors (LCAs) are depicted in the phylogenetic tree [59-61].

**Figure 5:**
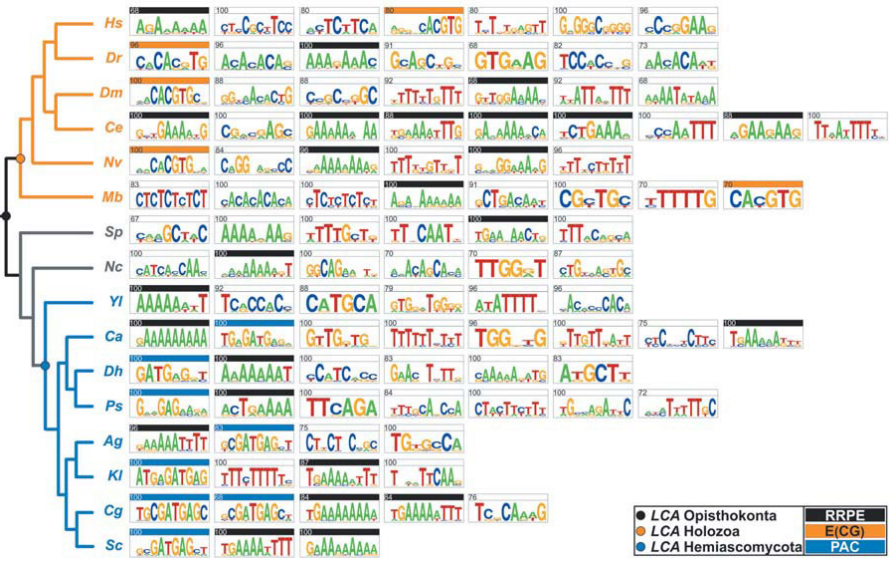
**Evolution of shared motifs in opisthokont RiBi core promoters**. The promoter gene sets for each species depicted in Figure 4 weres analyzed by MEME to identify common cis-regulatory motifs for each lineage [57,58]. Motifs found in greater than two-thirds of RiBi genes are depicted from left to right (highest scoring motifs to left). The frequency of each motif is expressed as a percentage in the upper left corner. The known fungal motifs RRPE [5,6] and PAC [5-7] are shown in shown in black and blue, respectfully. The holozoan E(CG) motif identified in this work is shown in orange.

Importantly, we also failed to find elevated levels of E(CG) in yeast RiBi versus control groups (*C*_1 _and *C*_*M *_in Fig. 2). Of relevance, the yeast RiBi genes are known to be regulated by motifs that are distinct from E(CG) [29-31]. We were also unable to find the E(CG)-RiBi signature in other fungal and more distantly related eukaryotic genomes (see Figs. 4, 5 and Methods).

Interestingly, there are a few fly E(CG)-bearing genes whose orthologs are E(CG)-bearing across humans, cnidarians, and choanoflagellates, but are not yet known to be related to ribosome biogenesis (Table 1). One of these is the *vasa *(DDX4) locus, which is an RNA-binding protein and metazoan germline determinant. This gene is maintained as an E(CG)-bearing promoter in humans, ascidians, flies, cnidarians, and choanoflagellates, but not in the nematode *C. elegans *and could conceivably be another DDX/DHX-containing RiBi gene, which was secondarily co-opted as a metazoan germline determinant. Another corresponds to the Dph5 gene, which is involved in the diphthamide modification of a histidine residue on elongation factor 2 (EF2) [32,33]. Dph5 is possibly required for frame-shift suppression during translation [34], but could conceivably play a role in unknown ribosomal modifications.

In contrast to the highly conserved nature of the RiBi regulon across Holozoa, there is little evolutionary conservation of E(CG) sites for genes not known to be involved in RiBi [see Additional file 1]. This indicates that these fly E(CG)-bearing genes, including some known Myc targets in flies and/or humans [e.g. CAD [35] and TIMM10 [36]], acquired the E(CG) site either in a stem bilaterian or in subsequent independent occurrences. These sites could also represent background noise due to sequence drift in a particular lineage. Many of these genes have fly E(CG) sites that are ± 600 bp of the initiation site, but are not as tightly linked to the core promoter (± 80 bp) as RiBi genes.

### The E(CG)-bearing RiBi regulon is found only in Myc-bearing genomes

Like the bilaterian genomes of flies and humans, the cnidarian *Nematostella*, a non-bilaterian metazoan, possesses Myc and Max homologs [27,37,38]. *Nematostella *Myc homologs can also bind to E(CG) in vitro as well as rescue the proliferative defects of myc null mammalian cells (Janice Ascano, S.J.B, and M.D.C.; in preparation). Intriguingly, the non-metazoan choanoflagellate genome of *Monosiga *also possesses clear orthologs of both Myc and Max (Fig. 3). However, outside of Holozoa, Myc and Max genes appear to be absent. Yeast do not possess Myc or Max orthologs even though E(CG)-binding bHLH dimers are present [39]. Moreover, the yeast RiBi genes, which lack E(CG) sites, are known to be regulated by non-bHLH factors [29-31,39]. We were also unable to find members of the Myc clade of genes in other fungal and more distantly related eukaryotic genomes (see Methods).

### Secondary loss of Myc is linked to secondary loss E(CG) RiBi signature in nematodes

Unlike flies and humans, the nematode represents a bilaterian that appears to have secondarily lost Myc but not Max (Fig. 3) [38]. This loss of a nematode Myc together with the loss of the E(CG) signature in the RiBi regulon is intriguing and suggests a few hypotheses and predictions. We explore these here because the loss of both an important metazoan transcription factor and a statistically significant association of E(CG) motifs with the holozoan RiBi gene battery is noteworthy and instructive of Myc function.

One major developmental hypothesis of nematode loss of both Myc and E(CG) sites in RiBi core promoters might stem from the relatively small number of 1000 somatic cells in the adult nematode [40]. This extremely low quantity of adult cells and limited cell-proliferation may render Myc's induction of the RiBi regulon unnecessary. Thus, under this hypothesis, the RiBi regulon is under two modes of regulation in Holozoa. First, there is a basal rate of low-level expression, and second there is a Myc-dependent induced rate of elevated expression that is associated with proliferating cells. Thus, nematodes with their particular small body sizes, and limited cell numbers, might have evolved to forgo Myc-RiBi induction. If this hypothesis is correct, we might expect to find further pseudogenization of the Myc locus, in animals with similar character. Other phyla known to include animals of such type include gastrotrichs, kinorhynchs, loriciferans, nematomorphs, and some priapulids.

An alternative molecular hypothesis of nematode-specific loss of both Myc and E(CG) sites in RiBi core promoters involves the nematode phenomenon of trans-splicing [41,42]. Nematodes employ a distinctive 5'-mRNA capping process that depends on trans-splicing of pre-capped 5' leader sequences expressed at independent loci. Because Myc has recently been found to upregulate mRNA capping of target genes [43,44], trans-splicing might have alleviated the need for keeping the Myc gene.

Yet a third hypothesis for loss of both Myc and E(CG) sites in RiBi core promoters involves novel shared motifs in *C. elegans *RiBi genes. Interestingly, as described further below our investigation analysis across opisthokont genomes, reveals several additional motifs resembling the RRPE motif that is present across Opisthokonts (Fig. 5). This could indicate a greater reliance on factors binding the RRPE motif or an augmented basal level of expression. Several studies have documented the evolution of cis-regulatory signatures via substitution of transcription factors that regulate even highly conserved gene sets [45,46].

All three hypotheses for loss of nematode *myc *along with loss of the E(CG)-RiBi signature are mutually non-exclusive. For instance, loss of proliferative cells and the underlying Myc genetic circuitry might have been permissive for the development of trans-splicing. Alternatively, the evolution of nematode trans-splicing might have occured first and been permissive for loss of Myc regulation.

### Mad and Mnt are dispensable for the E(CG)-RiBi core promoter signature

The loss of specific bHLH genes, such as the loss of Myc in nematodes, results in different bHLH repertoires present across holozoan genomes. We therefore next examined genomic bHLH repertoires among different organisms in order to consider potential trans-factors other than Myc that might correlate with E(CG) core elements in RiBi promoters (Fig. 3).

Among the Myc superfamily, Max:Max, Mad:Max, Mnt:Max, and Myc:Max dimers, can all bind to E(CG) [12,13,23]. The continued presence of both Mad and Max in nematodes [38] suggests that Mad:Max or Max:Max complexes do not target the RiBi regulon via the E(CG) target site, which is absent in nematode genomes (Fig. 3A). Furthermore, a Mad ortholog is not present in *Drosophila*, whereas both Mnt and Mad are apparently absent in *Monosiga *suggesting that both are dispensable for the function of the E(CG)-RiBi signature (Fig. 3A). [Both Mad and Mnt are present in other eumetazoan genomes such as cnidiarians (*N. vectensis*) and sea urchins (*S. purpuratus*), but are reciprocally lost in flies (no Mad) and nematodes (no Mnt) (Fig. 3A).]

All of these genomic configurations suggest that E(CG) promoter signatures are direct targets of Myc:Max complexes and are consistent with both the known biochemically confirmed RiBi targets of Myc as well as the growth-related phenotypes of *myc *mutant alleles. Nonetheless, these results do not exclude the possibility that other E(CG)-binding proteins potentially modulate the RiBi regulon when they coexist with Myc in the genome [47]. Thus, in conjunction with our RiBi-E(CG) genomic analyses, a consideration of the bHLH repertoire of holozoan genomes supports the hypothesis that the need for regulation by Myc, but not by other bHLH genes, is responsible for the evolutionary maintenance of E(CG) sites across the RiBi suite of genes.

### Evolution of RiBi core promoter sequences across Opisthokonts

While we were not able to find either Myc or Max orthologs in any available genome outside of Holozoa, it is still possible that the E(CG) motif is still associated with RiBi genes and controlled by other factors. For instance, *Saccharomyces cerevisiae *possesses two E(CG)-binding bHLH complexes from its small repertoire of bHLH genes [39]. These correspond to Pho2p/Pho4p heterodimers and Cbf1p homodimers. The Pho2p/Pho4p complex is involved in the regulation of phosphate biogenesis in responses to phosphate starvation, whereas Cbf in involved in centromeric function, methionine biosynthesis, sulfur metabolism, and regulating ribosomal structural proteins, [39,46]. We therefore looked for the presence of E(CG) in the core promoters of RiBi genes of 10 different fungal genomes (Fig. 4). We specifically looked at the 25 RiBi orthologs that are E(CG)-bearing across Holozoa (Table 1B). We find that the average rate of E(CG) sites across all identifiable RiBi orthologs in the 500 bp window immediately upstream of the start ATG (this window size corresponds to the typical intergenic distances in these fungal genomes) is 16%. Three separate fungal genomes have 0% E(CG)-bearing RiBi core promoters (0/25 RiBi promoters), while *Ashbya gossipyi *has the highest at 37.5% (9/24 RiBi promoters). By comparison, in the 500 bp core promoter window of the 25 RiBi genes in choanoflagellates the rate is 72% (18/25), while in flies it is 84% (21/25); the few missing promoters have E(CG) motifs in the adjacent upstream 100 bp. Thus, this core set of RiBi genes is not likely to be regulated by E(CG) motifs in fungi as the majority of their promoters lack this site.

We next conducted a MEME analysis of 500 bp core promoter fragments across multiple holozoan and fungal core promoter sequences for RiBi genes to identify all potential motifs that might serve as common binding sites in each system. We find that the previously identified PAC (Polymerase A and C) RiBi motif [5-7] can be readily identified in almost all Hemiascomycete fungi except the distantly related *Yarrowia lipolytica*. This motif, when present, is usually found in 100% of all RiBi genes analyzed in each species. Thus, the same genes that are likely co-regulated by Myc:Max via E(CG) in Holozoa are instead co-regulated by PAC binding factors in the Hemiascomycota sub-phylum. Interestingly, among the Myc targets identified in flies, we have found subunits of DNA-dependent RNA polymerases [see Additional file 1].

Unexpectedly, the RRPE motif, which was identified as a co-occuring motif in yeast RiBi genes along with PAC motifs, appears in both fungal and holozoan RiBi genes promoters. A yeast-specific factor, Stb3, has been proposed to promote cell growth by binding to at least some RRPEs in target genes in a glucose-dependent manner [48]. However, not all RRPE-bearing yeast promoters appear to require Stb3 for induction [48]. Nonetheless, this motif may represent a more ancient and possibly conserved RiBi binding motif than previously appreciated. In this case, Holozoan-specific and Hemiascomycete-specific modes of RiBi regulation might be relatively more recent additions since their divergence. Altogether, these analyses support the conclusion that E(CG) is uniquely associated with Myc-bearing Holozoan genomes and that different signatures and factors control the same regulon in distant taxa.

## Conclusion

We successfully identified a specific core-promoter signature, partially composed of the Myc:Max binding site E(CG), which is highly specific to the entire suite of genes devoted to ribosome biogenesis in Holozoa but not in fungal genomes. Based on these whole-genome analyses, and the confirmation of individual RiBi genes as Myc targets, we conclude that the entire RiBi gene set constitutes a bona fide Myc-targeted regulon. By analyzing a wide diversity of eukaryotic genomes, we show that this specific core-promoter signature is present only in holozoan genomes that still contain Myc. Furthermore, gene loss in other Myc:Max superfamily members, such as Mad or Mnt, while retaining Myc, is apparently not sufficient for loss of the E(CG) signature in the RiBi gene set. Thus, nematode genomes, which lack Myc, but not Max or Mad, do not possess the E(CG) signature across the well-conserved RiBi gene set.

A toolkit of animal-specific genes, including the bHLH family of DNA-binding factors, is thought to have been assembled early in pre-animal evolution. The bHLH family has been intriguing because of its diversification into cell-type specific functions modulating proliferation, differentiation, and metabolic programs across eukaryotes [37-39,49,50]. Consequently, the evolution of animals is likely to have involved the establishment of a canonical set of bHLH transcription factors regulating these downstream genomic programs. Here, we describe a large RiBi regulon that co-evolved with Myc:Max in a stem-holozoan. Significantly, Myc and Max mark the beginning of an animal-like bHLH repertoire in a pre-metazoan ancestor.

Choanoflagellates represent the sister-group to animals. Their ability to form colonies is indicative of a possible precondition in the evolution of multicellularity in metazoa [51]. Furthermore, at least one Receptor Tyrosine Kinase (RTK) has been identified in choanoflagellates [52]. RTKs were once thought to be exclusive to animals, which use them in cell-cell communication and as signaling inputs into growth factor-mediated pathways of gene activation such as Myc [53]. These results suggest a model for the origins of a Myc-induced RiBi regulon, which is commonly misregulated in diverse human cancers. Around 750 to 1000 million years ago [54], a protozoan, heterotrophic ancestor of Holozoa either adapted, or co-opted through duplication and divergence, a proto-Myc:Max bHLH heterodimer complex and evolved the capability to induce the primordial Myc moiety in response to RTK-mediated growth signals. Core promoter-bound Myc:Max complexes would then co-ordinately up-regulate the ribosome biogenesis regulon and thereby commit to cell growth and/or proliferation.

An alternative hypothesis would be that the RiBi genes have always been inducible in diverse taxa, but that this mode of regulation has diverged and/or been supplanted by distinct mechanisms in different taxa. Thus, the Holozoan E(CG) RiBi signature would not represent a new ability to up-regulate this gene set but rather a unique mechanism for inducing the RiBi gene cohort. Similarly, in the hemiascomycete fungi (except for the distantly related *Yarrowia*) the RiBi gene set is co-regulated by non-bHLH factors via the PAC motif.

A recent study has also identified E(CG) as present in a subset of core promoters of yeast ribosomal structural protein-encoding genes driven by Cbf1p [46]. Yeast are well known to have at least two E(CG)-binding bHLH systems in Pho2/Pho4p and Cbf1p complexes, which are involved in phosphate biogenesis and methionine biosynthesis/translation, respectively. We also see a slight elevated level of E(CG) in some fungal genomes such as *Pichia *and *Ashbya*. Thus, the Holozoan Myc and Max system might have evolved out of an Opisthokont ancestor in which E(CG) motifs might have been loosely tied to generic growth programs controlling both RiBi and RP gene sets. Under this scenario, a general cell growth pathway culminating in a bHLH induction of an undefined regulon might have existed in an ancient opisthokont ancestor. This pathway then diverged separately in fungi and Holozoa. In Holozoa, this bHLH gene evolved into a bHLH-ZIP encoding gene and subsequently duplicated and diverged to produce Max, and a growth-inducible activating form with Myc. The Myc-Max then specialized in induction of RiBi genes. In fungi, this system perhaps retained its ancestral homodimeric form in Cbf1p and controlled a more generic cell growth pathway that included ribosomal proteins, a few RiBi genes, as well as other cell growth functions. Furthermore, the RiBi genes in hemiascomycetes evolved to be largely controlled specifically by PAC-binding factors. Future research will have to be conducted in both fungal and animal genomes to explore these ideas.

## Methods

### Statistical analysis

Statistical tests were conducted to test for the significance of the difference between RiBi, mitochondrial (*C*_*M*_), and other control (*C*_1 _or *C*_2_) gene sets. A Mann-Whitney test for the significance of the difference between RiBi genes and control orthologs (*C*_1 _or *C*_2_, see Fig. 2) was statistically significant (*p *< 0.001) for Hs, Dm, Nv, and Mb data. There was no statistical significance between RiBi orthologs and control data for Ce or Sc. A Mann-Whitney test for the significance of the difference between non-RiBi genes with RiBi-type promoters, and mitochondrial or control orthologs (*C*_1 _or *C*_2_, see Fig. 2) was statistically significant (*p *< 0.001) for Hs and Dm data. However, there was no statistical significance between RiBi-type promoter bearing non-RiBi gene orthologs and control data or mitochondrial orthologs for Nv, Mb, Ce, or Sc data. Statistical validation of over-represented GO terms shared by genes that we identified in Fig. 1 was carried out as previously described [55]. The GO term "Ribosome Biogenesis and Assembly" was found to be statistical significant (*p *< 1.2*e *- 27) based on Fisher's Exact Test (one-sided P-value) of the association between attribute and query.

### Human and fly orthology

Lists of fly and human genes together with their corresponding DNA sequences (± 600 bp from 5'-annotated end) meeting orthology gene tree tests between genomes were retrieved from Ensembl data mining tool BioMart. The annotation data corresponded to Ensembl Release 44, 2007 using genome builds *Drosophila melanogaster *BDGP 4.3 and *Homo sapiens *NCBI 36. This resulted in 3066 human/fly pairs although some groups of pairs correspond to multiple isoforms in one genome. DNA sequences were searched for CACGTG E-boxes [E(CG)] using the UNIX grep and perl tools. Orthologous gene pairs with E(CG) in each genome were compared with a list of verified ribosome biogenesis (RiBi) genes [56], the list of yeast orthologs with "nucleolar" GO classification, and current results in the literature (Pubmed).

### Genome assemblies and orthology identification

We used the following genome builds: *Drosophila melanogaster *BDGP 4.3, *Homo sapiens *NCBI 36, *Saccharomyces cerevisiae *SGD1.01, and *Caenorhabditis elegans *WS170. We used Ensembl Release 44 (2007) for orthology calls between these four genomes. ENSEMBL orthology calls use best reciprocal hits between genomes to cluster proteins followed by construction of maximum likelihood phylogenetic gene trees (NJTREE) and distinguish orthologs from paralogs. For identifying orthologous loci in the *Nematostella vectensis *and *Monosiga brevicollis *we used BLASTP to identify the best matches in the respective genomes to the *Drosophila *amino acid sequence. EST coverage in each genome allowed independent confirmation of the majority of homologous sequences. We identified all hits with *E *<*e *- 10. We then performed a reciprocal BLASTP to weed out 1-to-many hits.

### Promoter sequence analysis

In searching for core promoter linked E(CG) sites we looked used the BDGP assembly release 4, flybase annotation rel.4.3-20060130. To identify potential E(CG) flanking patterns, the sequences adjacent to E(CG) sites in the conserved human/fly RiBi genes as well as other known confirmed Myc targets were aligned to identify the reported information content in the immediate flanking sequences. Additionally, these sequences were compared to a variety of control sequences composed of anti-signature 2 motifs, promoter sequences of adjacent genes, or unrelated developmental genes (anterior/posterior *Drosophila *developmental loci) to identify over-represented motifs spanning 6 to 8 bp with 0, 1 or 2 wild cards. This resulted in the identification of three classes of motifs: 1) E(CG) with flanking sequence, 2) DRE sequences, 3) A-rich sequences (Fig. 1). Genome queries were conducted by direct searches of the most recent *Drosophila *genome (BDGP 4.3) using UNIX grep and perl. Genomic queries for signatures were defined as follows. Signature 1 identifies 15,434 in the *Drosophila *BDGP 4.3 genome searches. Signature 2 was any sequence matching one of the following CAACACGTGCG, AAACACGTGTG, and AAACACGTGCG. Signature 3 was defined as a window of 80 bp containing CACGTG and 2 of the following sequences: CTATCG or TATCGA. Loci that mapped within 600 bp, 240 bp or 1 kb of these three signatures, respectively, were identified.

### bHLH phylogenetic analyses

Over 150 bHLH amino acid sequences from plant (*Arabidopsis thaliana*), ciliate (*Tetrahymena thermophilia*), yeast (*Saccharomyces cerevisiae*), choanoflagellate (*Monosiga brevicollis*), sponge (*Amphimedon queenslandica*), cnidarian (*Nematostella vectensis*), protostomes (*Drosophila melanogaster *and *Caenorhabditis elegans*) and deuterostome (*Strongylocentrotus purpuratus*) organisms were aligned using CLUSTALW and used to make a primary alignment and phylogenetic guide tree. Alignments were adjusted by hand to reduce the number of insertions, and were subsequently used to generate random samples using PHYLIP Seqboot. Phylogenetic trees were generated using neighbor-joining (PHYLIP Neighbor), parsimony (PHYLIP Protpars), or maximum liklihood (PHYLIP Proml). The analyses were conducted by using all, or diverse subsets of bHLH sequences, all of which supported the Myc and Max clades. The Mnt/Mad clades alternatively group with a Myc/Max clade or to the Max clade with a Myc sister clade to that. Choanoflagellate bHLH sequences were identified by BLASTP to bHLH sequences from yeast and *Drosophila*. This process identified 3 choanoflagellate bHLH sequences (Fig. 3).

### Genome conservation

Human, *Drosophila*, and *Saccharomyces *genomic data were retrieved for RiBi orthologs from Ensembl using their 1-to-1 orthology classification. *Nematostella*, and *Monosiga *single best BLAST matches (*p *< 10 - 5) were identified from the Joint Genome Institute data sets annotated by EST sequences and their genomic sequences retrieved. To ascertain that the absence of the E(CG) regulon in the yeast genome was not a secondarily derived trait akin to nematodes, we investigated other fungal genomes as well as more distantly related eukaryotic genomes, including at 10 other sequenced fungal genomes (JGI), as well as the ciliated protist *Tetrahymena thermophilia*, the single celled green alga *Chlamydomonas reinhardtii *(JGI), and the poplar tree *Populus trichocarpa *(JGI). Each genome was searched for BLASTP alignments using the fly Myc and Max bHLH amino acid sequences. Only the *Tetrahymena *genome produced a match, which upon various CLUSTALW alignments falls outside of the Group B superclade from which the Myc and Max clades group.

### Control Sequences

Control genes (*C*_1_) were obtained by finding neighboring downstream genes preserving orthology [see Additional file 2]. First, yeast control genes were identified by finding for each gene with a nucleolar GO term, the next downstream gene with an ortholog in the human genome. Orthology with a human gene ensured that control sequences were derived from genes that are as conserved as RiBi genes. Fly and worm control genes were generated by finding for each human RiBi ortholog, the nearest downstream gene a human ortholog. *Monosiga *and *Nematostella *downstream controls (*C*_1_) were generated by finding the nearest downstream EST to the corresponding RiBi gene. The presence of one or more E(CG) sites in the region ± 600 bp from the 5'-most annotated end was ascertained for each such control gene. Additional control sequences (C2) were obtained for the *Nematostella *and *Monosiga *genomes by taking ± 600 bp from the 3' annotated end of each test gene [see Additional file 1].

### MEME analysis and sequence logos

A set of RiBi orthologs meeting orthology gene tree tests between genomes were retrieved from the Ensembl data mining tool BioMart for Hs, Dr, Dm, Ce, and Sc genomes respectively. The annotation data used genome builds Hs (NCBI 36), Dr (Zv7), Dm (BDGP 5.4), CE (WS180) and Sc (SDG1.01). For identifying orthologous loci in Nv, Mb, and Ps, we used best reciprocal hits to identify orthologs in the respective genomes of the closest related species. A set of RiBi orthologs for Ag, Nc, and Sp were retrieved from the *Ashbya *Genome Database project base on Ensembl release 40. For identifying orthologous loci in Kl, Dh, Cg, Ca, and Yl we used ortholgy calls from the Génolevures Genomic Project. The DNA sequences for each species RiBi gene promoters were then collected from either Ensembl, JGI, or Génolevures for each respective genome. For fungal and choanoflagellate RiBi promoter sequences, -500 bp from the translational start site was collected for each ortholog. For Dm, Ce, Nv, Dr, and Hs, ± 250 bp from the 5' annotated end was collected for each ortholog. Each organism's RiBi sequence group was analyzed my MEME to determine overrepresented motifs [57,58]. Motifs 6–10 bp in length that occured either zero or one time in each sequence of each set per species such that at least 10–75 motifs per set were identified. Furthermore, a secondary cut-off stipulating that all motifs be found in at least 2/3 of all sequences per set was applied. Sequence logos from the matrices of over-represented motifs derived from MEME were then created using WebLogo.

## Authors' contributions

SJB, MDC, and AJE conceived and designed the bioinformatic and analytic strategies; AJE worked on the fly signature and whole fly genome queries; SJB worked on documenting the presence of the E(CG) signature in RiBi and control gene groups across eukaryotic genomes; both SJB and AJE made the final figures and tables; SJB, MDC, and AJE contributed to the writing and discussion of the paper.

## Supplementary Material

Additional file 1 **Catalog of E(CG)-bearing promoters by genome and gene function**. All fly genes matching the fly RiBi promoter architecture (see Methods), or else constituting the fly ortholog to a yeast nucleolar or RiBi gene is listed. For each such fly gene, the corresponding ortholog (human, nematode, and yeast) or closest 1-to-1 homolog (cnidarian, choanoflagellate) is listed along with the presence (1, red) or absence (0) of E(CG) within 600 bp of the annotated start site. The E values scores for BLASTP matches in the *Nematostella *and *Monosiga *genomes are given in parentheses for all matches with E <*e *- 5. Matches below e-10 are highlighted in yellow. Boxes containing N/A indicate the presence of the cis-element could not be addressed because a distinct 1-to-1 ortholog could not be identified. Red highlighted yeast genes were listed as RiBi genes in a recent comprehensive review of eukaryotic ribosome biogenesis [56]. C2 control data sets are shown where applicable. Click here for file

Additional file 2 ***C*_1 _control data sets for Figure 2**. The presence (presence = "1", red) or absence (absence = "0") of promoter-proximal (± 600 bp) E(CG) sites within downstream control genomic loci (*C*_1_) is indicated. Click here for file
